# A single-nucleotide variant conditions the ability vs. inability of *Propionibacterium freudenreichii* to utilize L-lactate

**DOI:** 10.1128/aem.00599-25

**Published:** 2025-06-12

**Authors:** Riccardo Cocuzzi, Meral Turgay, Remo S. Schmidt, Ueli von Ah, Hans-Peter Bachmann, Laure Weisskopf, Marie-Therese Fröhlich-Wyder

**Affiliations:** 1 Agroscope 419060, Bern, Switzerland; 2 Department of Biology, University of Fribourg 27211, Fribourg, Switzerland; Universita degli Studi di Napoli Federico II, Portici, Italy

**Keywords:** *Propionibacterium freudenreichii*, Swiss-type cheese, lactate metabolism, adaptive laboratory evolution, *lutABC*

## Abstract

*Propionibacterium freudenreichii* (*P. freudenreichii*) has various biotechnological applications, notably in the ripening of Swiss-type cheese, where it utilizes the two enantiomers of lactate as the main carbon source, contributing to flavor development and eye formation. Here, we genotypically characterized two *P. freudenreichii* strains unable to catabolize L-lactate through whole-genome sequencing and variant calling, using *P. freudenreichii* FAM-14222 as the reference genome, which highlighted a mutation in the *lutB* gene in both strains. This gene is part of the *lutABC* operon, which has been previously linked to lactate utilization in other bacterial species. Subsequently, we successfully restored the strains’ ability to utilize L-lactate by following an adaptive laboratory evolution approach, which involved repeated subculturing in a medium containing L-lactate as the main carbon source. Sequencing of the *lutB* gene confirmed that isolates with a restored ability to utilize L-lactate had also reverted the mutation back to wild-type, supporting the involvement of the *lutABC* operon in L-lactate catabolism in *P. freudenreichii*. Moreover, the phenotype of the two L-lactate-negative strains was confirmed under cheesemaking conditions, highlighting the potential of the strains as cheese ripening cultures.

**IMPORTANCE:**

Lactate catabolism is of paramount importance in *Propionibacterium freudenreichii*, particularly for its industrial applications, such as Swiss-type cheese ripening. Nevertheless, the genetic background of this metabolic process is not fully understood. In our study, we developed an adaptive laboratory evolution-based approach for the elucidation of L-lactate catabolism, starting from two strains unable to utilize L-lactate. Our results delivered experimental evidence of the role of the *lutABC* operon in this process, as opposed to the widespread theory of L-lactate dehydrogenase-mediated oxidation. A deeper understanding of this metabolic pathway will be beneficial for a more efficient selection of industrial strains, as well as for metabolic engineering.

## INTRODUCTION

*Propionibacterium freudenreichii* (*P. freudenreichii*) is a Gram-positive, non-motile, pleomorphic bacterium with high GC content. It has various biotechnological applications, ranging from cheese ripening to industrial production of vitamin B12 and probiotic activity. Reported beneficial properties of *P. freudenreichii* include microbiota modulation, immunomodulation, and the induction of apoptosis of colorectal carcinoma cells due to the release of short-chain fatty acids (1–3). Experimental cheese exclusively fermented with *P. freudenreichii* was found to contain immunomodulatory proteins, and its consumption protected mice from acute colitis (4). The ability of *P. freudenreichii* to ferment DL-lactic acid is a key factor to ensure its growth in Swiss-type cheeses (5). During Swiss-type cheese manufacturing, lactose is fermented by starter lactic acid bacteria (LAB) (*Streptococcus thermophilus* and *Lactobacillus delbrueckii* subsp. *lactis*) to L- and D-lactic acid in a ratio of approximately 1:1 within 24 hours from the beginning of cheesemaking (6). To slow down the ripening process by inhibiting the activity of *P. freudenreichii*, facultatively heterofermentative Lactobacilli (*Lacticaseibacillus casei* and *Lacticaseibacillus rhamnosus*) may be added to the starter LAB. However, this addition generally yields cheese with slightly poorer sensory properties (7). After lactic acid fermentation, the metabolic activity of *P. freudenreichii* is responsible for the development of the typical sweet and nutty flavor of this cheese variety, and for the so-called eye formation, where cavities are formed along the ripening process by the production of CO_2_ (8). In addition to lactate fermentation, amino acid catabolism in *P. freudenreichii* enables the production of various volatile compounds, such as isovaleric acid, which contribute to flavor development (9). It is commonly accepted that in the metabolic pathway involved in lactate catabolism, DL-lactate is converted to pyruvate, which is further oxidized to acetate and CO_2_ or reduced to propionic acid via the Wood-Werkman cycle, which uses the NADH deriving from glycolysis, from the conversion of DL-lactate to pyruvate or from the conversion of pyruvate to acetate (6). The conversion of DL-lactate to pyruvate in *P. freudenreichii* has been described in several reports as catalyzed by DL-lactate dehydrogenases (5, 10–12). However, the function of these enzymes has been recently linked to lactate production, rather than consumption (13). Moreover, as observed by Turgay et al. (14), the presence of a D-lactate dehydrogenase in the genome is not required for *P. freudenreichii* to metabolize D-lactate under anaerobic conditions, suggesting the presence of uncharted, alternative pathways for the oxidation of lactate to pyruvate in this species.

Despite its importance in Swiss-type cheese ripening, lactate degradation in *P. freudenreichii* is still poorly characterized. In this study, we developed an adaptive laboratory evolution (ALE)-based approach for the elucidation of the genetic background of L-lactate catabolism in *P. freudenreichii*. First, two *P. freudenreichii* strains originating from the Agroscope strain collection, which were unable to catabolize L-lactate (L-lactate-negative), were phenotypically characterized for their behavior during the ripening of Swiss-type model cheese. The two strains were then genotypically characterized, highlighting mutations of particular interest for the explanation of the L-lactate-negative phenotype. Subsequently, the two strains were cultured in an L-lactate-containing medium, allowing for the selection of mutations providing the strains with a gain of fitness, which restored the L-lactate-utilizing phenotype in both strains. Finally, Sanger sequencing confirmed the correlation between the presence of a specific single-nucleotide polymorphism and the strains’ ability to catabolize L-lactate.

## MATERIALS AND METHODS

### Production of model Swiss-type cheese

Three variants of model Swiss-type cheese were produced at the Agroscope pilot plant (Bern, Switzerland), by modifying a previously published protocol (15). Pasteurized milk (90 L), standardized to a fat content of 35 g/kg, was supplemented with water (8 L) and heated to 31°C. Powdered hay (1 mg) was added to the milk as a source of nuclei for eye formation, as previously described (16). The milk was inoculated with 2‰ (vol/vol) of a liquid starter culture containing strains of *Streptococcus thermophilus* and *Lactobacillus delbrueckii* subsp. *lactis* (RMK 101, Liebefeld-Kulturen AG, Bern, Switzerland). Pre-ripening was carried out at 32°C for 30 min, then the milk for the three different variants was inoculated with *P. freudenreichii* FAM-3974, *P. freudenreichii* FAM-3981, and with a commercial culture of *P. freudenreichii* (Prop 01, Liebefeld-Kulturen AG), respectively. Table 1 summarizes the ripening cultures used, as well as their lactate utilization phenotypes. The concentration of *P. freudenreichii* in the milk was set to approximately 10^4^ CFU/mL, according to the specifications of the commercial culture and the long-term experience of cheesemakers in practice and research with this culture. Liquid cultures of the two L-lactate-negative strains were diluted in saline solution (sodium chloride [Merck, Darmstadt, Germany] 8 g/L, casein peptone [Merck, Darmstadt, Germany] 1 g/L), to match the concentration of the commercial culture. Upon the addition of approximately 18 mL of rennet (Winkler GR orange, Winkler AG, Konolfingen, Switzerland), the milk coagulated in approximately 35 minutes, after which the coagulum was cut into grains measuring about 3–5 mm within 15 minutes. Water (1 L) was then added, and the mixture of curd grains and whey was heated to 53°C over 30 minutes, scalded at the same temperature for 35 minutes, transferred into a mold, pressed with controlled cooling (53°C → 50°C, 2 h; 50°C → 35°C, 5 h; 35°C, 10 h; 35°C → 25°C, 1 h), and brine-salted (12°C, 16 hours). The cheeses, measuring 30 cm in diameter and weighing between 6 and 7 kg, were initially stored at 12°C for 10 days, then transferred to a warm room (22°C, 80% relative humidity) for 60 days, and finally ripened in a cool room (12°C, 70% relative humidity) until reaching 240 days of age. Cheese maturation took place in plastic bags for better humidity control. A second production run was performed as a replicate in the same week, following the same procedure as described above. At the ages of 1, 60, 120, and 240 days, D- and L-lactic acid in the cheese were determined using commercial enzymatic assay kits (R-Biopharm AG, Darmstadt, Germany).

**TABLE 1:** *P. freudenreichii* cultures used for model Swiss-type cheese production

*P. freudenreichii* culture	Lactate-utilizing phenotype	Source
FAM-3974	L-lactate-negative	This study
FAM-3981	L-lactate-negative	This study
Prop 01	DL-lactate-positive	Liebefeld-Kulturen AG

### Genotypical characterization of L-lactate-negative *P. freudenreichii*

Two *P. freudenreichii* strains unable to utilize L-lactate, namely FAM-3974 and FAM-3981, were sourced from the Agroscope strain collection. The genomes of the two strains were sequenced with the Ion Torrent short-read technology. After sequencing, the bioinformatics pipeline started with a quality control of raw reads with FastQC version 0.11.9 (17), then the reads were trimmed with Trimmomatic (18) (Trimmomatic settings: HEADCROP:6 CROP:400 SLIDINGWINDOW:4:20 MINLEN:50) and aligned with bwa mem version 0.7.17 (19) to the sequence of *P. freudenreichii* FAM-14222 (GenBank accession number CP191348), a closely related strain that shows no impairment in the utilization of L-lactate. The closeness of the relationship of FAM-14222 to the two L-lactate-negative strains was determined via whole-genome average nucleotide identity (ANI), calculated with FastANI (20) by comparing the two strains to the database of the Agroscope strain collection (FAM-3974 vs FAM-14222, ANI = 99.9890; FAM-3981 vs FAM-14222, ANI = 99.9958). Variants were called on bam files using bcftools mpileup version 1.9 (21). To predict the biological impact of the variants, the resulting vcf files were annotated with SnpEff version 5.1d (22), running with a database based on *P. freudenreichii* CIRM-BIA1.

### Adaptive laboratory evolution

**Fig 1:**
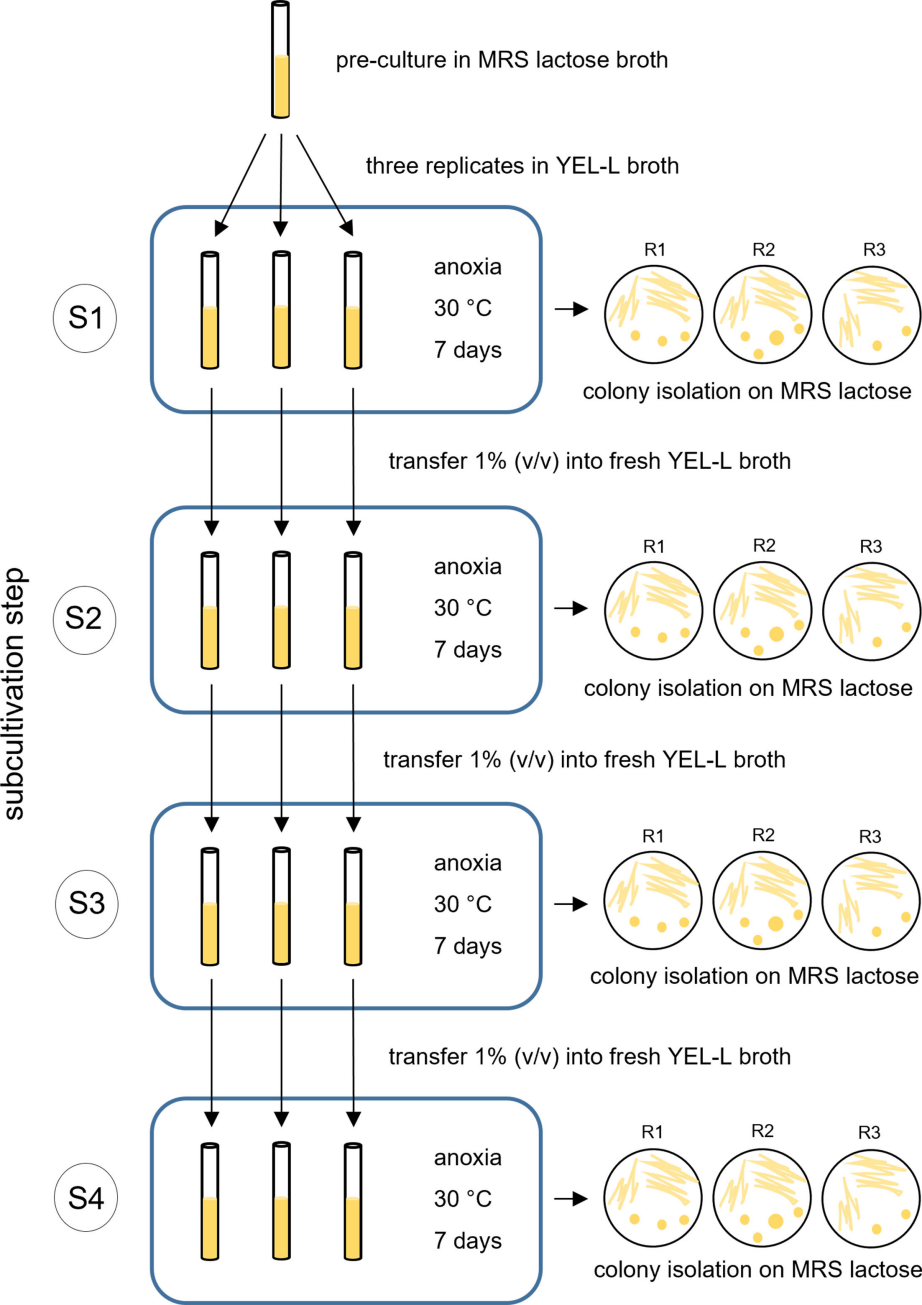
Experimental design for adaptive laboratory evolution. S1–S4: subcultivation steps; R1-R3: biological replicates. Each tube represents one biological replicate. For further details, see “Adaptive laboratory evolution,” above.

The restoration of the L-lactate-utilizing phenotype in L-lactate-negative *P. freudenreichii* strains was promoted by prolonged cultivation in a medium containing L-lactate as the main carbon source. In detail, the strains were pre-cultivated in MRS-lactose broth (Biolife, Milan, Italy) for 72 hours at 30°C under anoxic conditions. Anoxic conditions were generated with GENbox Anaer generator sachets (BioMérieux, Lyon, France), placed in a sealed polycarbonate culture jar (GasPak Anaerobic System, Becton Dickinson AG, Switzerland) (23) in all experiments. Each pre-culture was inoculated 1% (vol/vol) into three tubes, each containing 10 mL of Yeast Extract L-lactate (YEL-L) broth, a modified version of YEL broth (24) prepared with 115 mM sodium L-lactate instead of sodium DL-lactate (Merck, Darmstadt, Germany). The tubes were incubated for seven days at 30°C under anoxic conditions (subcultivation step 1), then the cultures were transferred 1% (vol/vol) into fresh YEL-L broth, and the incubation step was repeated for a further 7 days (subcultivation step 2). This procedure was reiterated until 28 days had elapsed since the beginning of the experiment (subcultivation steps 3 and 4). Samples for stock cultures, which were stored at −80°C after the addition of 20% (vol/vol) glycerol (Merck, Darmstadt, Germany), were taken after each incubation step. At the same time points, 1 µL/loop of the culture was streaked on MRS-lactose agar, allowing subsequent isolation of a single colony for further characterization (Fig. 1). The plates were incubated at 30°C for 5 days under anoxic conditions. As a control, the same experiment was repeated using YE broth (YEL-L broth with no added sodium L-lactate) instead of YEL-L broth for the ALE procedure.

### L-lactate utilization assays

Lactate utilization was evaluated by cultivating the isolates from each subcultivation step and biological replicate in a medium containing L-lactate as the main carbon source. For this purpose, single colonies of the isolates were pre-cultivated in MRS lactose broth for 72 hours at 30°C under anoxic conditions. The culture was washed in saline solution (sodium chloride [Merck, Darmstadt, Germany] 8 g/L, casein peptone [Merck, Darmstadt, Germany] 1 g/L) and inoculated 1% (vol/vol) in YEL-L broth. After incubating for 72 hours at 30°C under anoxic conditions, the biomass was pelleted (4,000 g, 25°C, 10 min) (Rotanta 460R, Hettich, Bäch, Switzerland), and the supernatant was stored at −20°C for lactic acid determination. D- and L-lactic acid were determined using commercial enzymatic assay kits (R-Biopharm AG, Darmstadt, Germany).

### DNA extraction and Sanger sequencing

*P. freudenreichii* isolates were cultivated in MRS lactose broth under anoxic conditions at 30°C for 72 h. For DNA extraction, a modified protocol by Turgay et al. (25) was followed. Briefly, cell pellets were resuspended in 200 µL of enzymatic lysis buffer (DNeasy Blood and Tissue Handbook 07/2006, QIAGEN AG, Hombrechtikon, Switzerland) containing 25 mg/mL of lysozyme and were incubated at 37°C for one hour; then, 10 µL of proteinase K was added (EZ1 DNA Tissue Kit, QIAGEN), and the samples were incubated at 56°C for 1 hour. DNA purification was completed using the BioRobot EZ1 workstation (QIAGEN) according to the manufacturer’s instructions, with an elution volume of 100 µL. For Sanger sequencing, the *lutB* gene was amplified by high-fidelity PCR using the following mixture: 0.5 µM of each primer (lutB_F: 5′-ATGAGCACCGAACTGCGT-3′; lutB_R: 5′-TTACCTCTCGTCCTCAACGC-3′), 1× SuperFi GC Enhancer, 1× Platinum SuperFi II PCR Master Mix (Invitrogen, Thermo Fisher Scientific, Waltham, MA, USA). Reaction conditions were set as follows: initial denaturation at 98°C for 30 s, followed by 35 cycles of 98°C for 10 s, 61°C for 10 s, 72°C for 45 s, and a final step at 72°C for 7 min (MiniAmp thermal cycler, Applied Biosystems, Waltham, MA, USA). The PCR products were purified using the QIAquick PCR Purification Kit (Qiagen, Hilden, Germany) and sequenced by a commercial Sanger sequencing service (Fasteris, Plan-les-Ouates, Switzerland).

## RESULTS

### DL-lactate degradation during model Swiss-type cheese maturation

**Fig 2:**
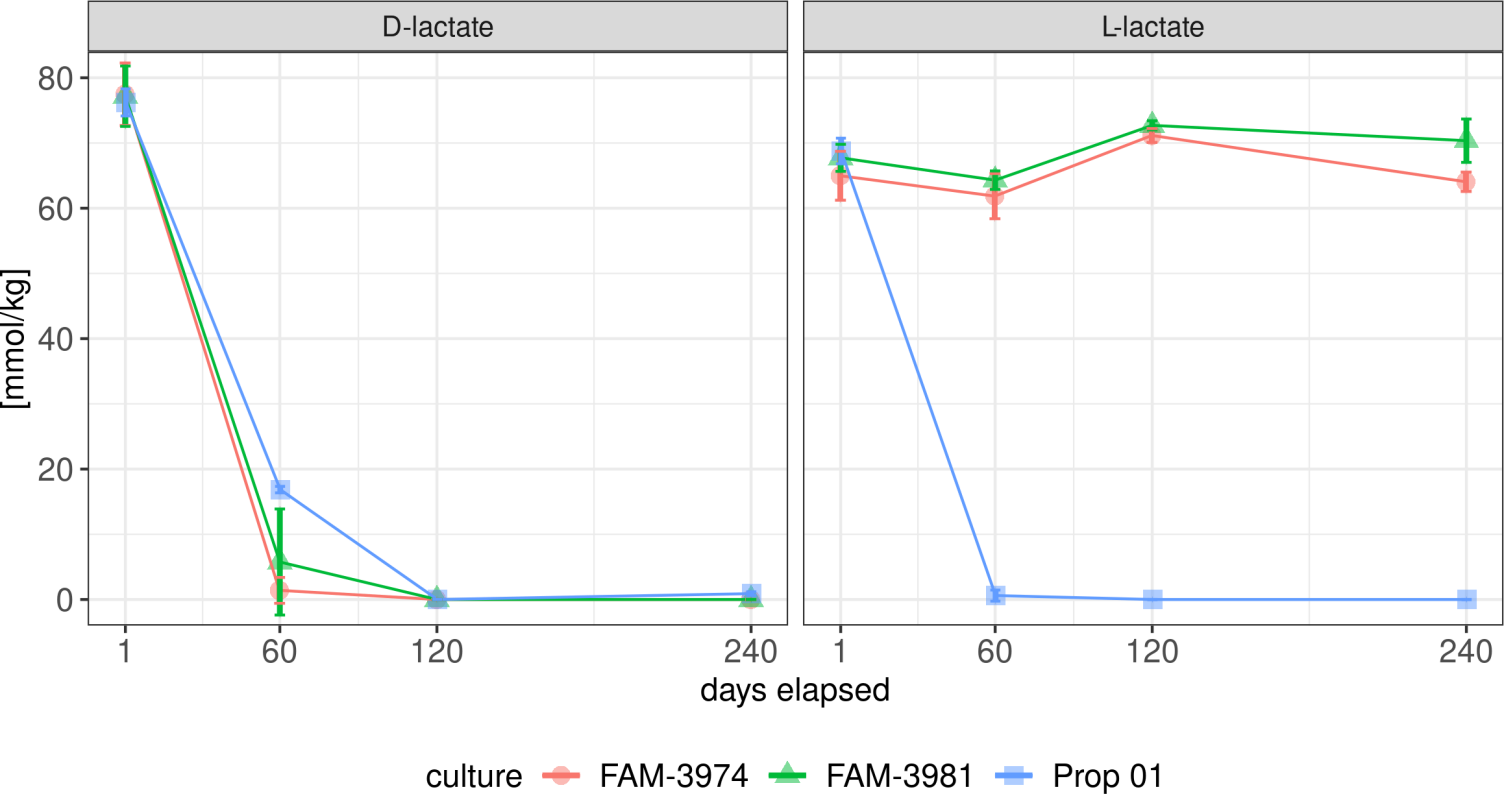
Residual DL-lactate in model Swiss-type cheese. Values on the y-axis represent means calculated with *n* = 4 for each time point, except 60 days. For 60 days, *n* = 2. Error bars depict standard deviation.

To test the L-lactate-negative phenotype of FAM-3974 and FAM-3981, model Swiss-type cheese was produced, using the two strains as ripening cultures, as well as Prop 01, a commercial culture of *P. freudenreichii* strains (Liebefeld-Kulturen AG, Bern, Switzerland) as a control. For all three cultures tested, D-lactate was consumed completely within the first 120 days of maturation. L-lactate was not degraded until the end of maturation in the cheeses produced with FAM-3974 and FAM-3981, while being completely depleted after 60 days in the control (Fig. 2). In the control, L-lactate was depleted more quickly than D-lactate (Welch’s unequal variances *t*-test at 60 days of maturation, *P*-value = 0.004965. Test run on R version 4.3.3); after 60 days, 99.1% of the L-lactate initially present in the curd had been depleted, while 77.9% of D-lactate had been consumed. DL-lactate measurement during cheese ripening highlighted the enantioselective phenotype of the two strains object of this study.

### Genotypical characterization of L-lactate-negative *P. freudenreichii* strains

**Fig 3:**
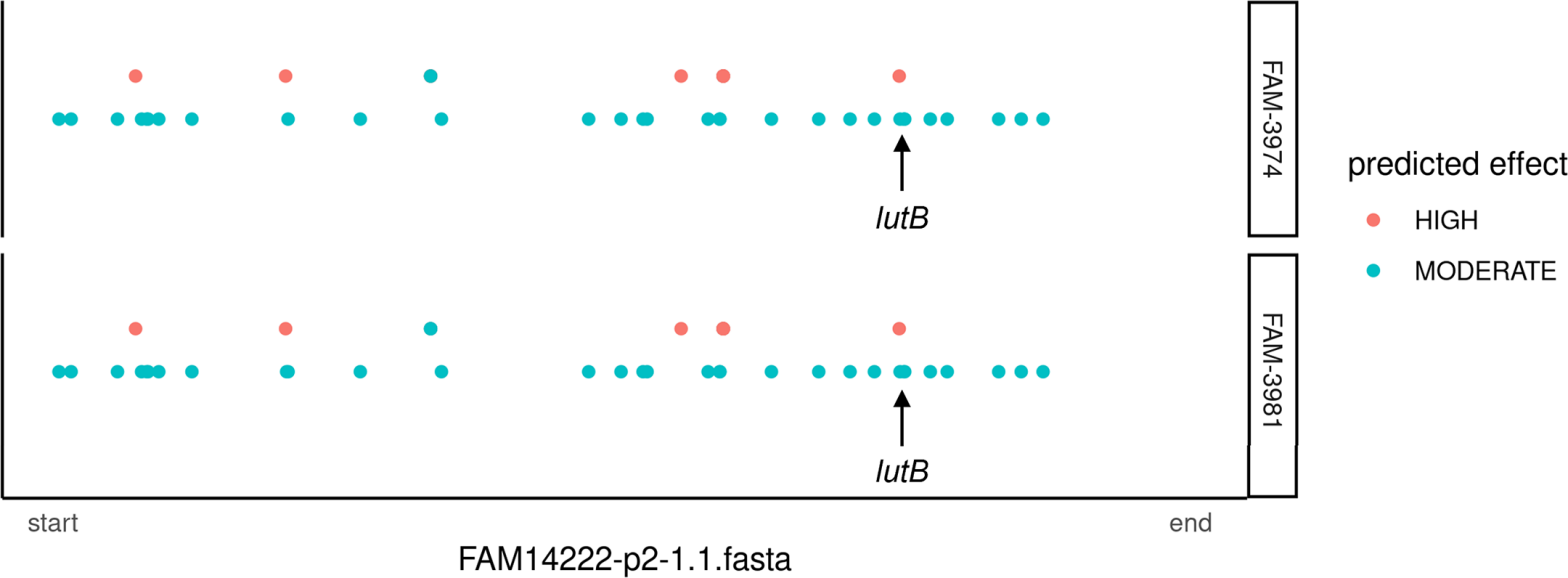
Visualization of variants found in L-lactate-negative strains with moderate and high impact, as predicted by snpEff. Each dot represents one variant found in the two strains of interest along the genome of FAM-14222, classified by type.

Short reads originating from the two L-lactate-negative strains FAM-3974 and FAM-3981 were mapped on the sequence of the closely related strain FAM-14222, which can metabolize both L- and D-lactate, to identify variants potentially explaining the L-lactate-negative phenotype. Variant calling highlighted the presence of 91 variants in FAM-3974, 63 of which were single-nucleotide variants (SNVs), and 28 insertions or deletions (indels). In FAM-3981, 87 variants were found, of which 64 were SNVs and 23 were indels. Figure 3 shows an overview of the variants with a moderate or high biological impact, as predicted by SnpEff, found in the two L-lactate-negative strains and their positions along the reference genome. Among the variants that were estimated to be non-silent by SnpEff (Table S1), an SNV in the *lutB* (lactate utilization protein B, locus tag FAM14222-p2-1.1_001787) was of particular interest for further analyses due to its putative function. The position of the variant in *lutB* is highlighted with an arrow in Fig. 3. Interestingly, no variant was detected in the two annotated L-lactate dehydrogenases, which are commonly regarded as the key enzymes for the oxidation of L-lactate to pyruvate (5, 10, 11). A total of 48 non-silent variants were found to be shared between the two L-lactate-negative strains. Among others, two variants were found in genes annotated as hypothetical proteins, one variant in a DUF domain-containing protein, and three variants in genes annotated as transporters.

### Restoration of the L-lactate-utilizing phenotype in L-lactate-negative *P. freudenreichii* strains and sequencing of the *lutB* gene

**Fig 4:**
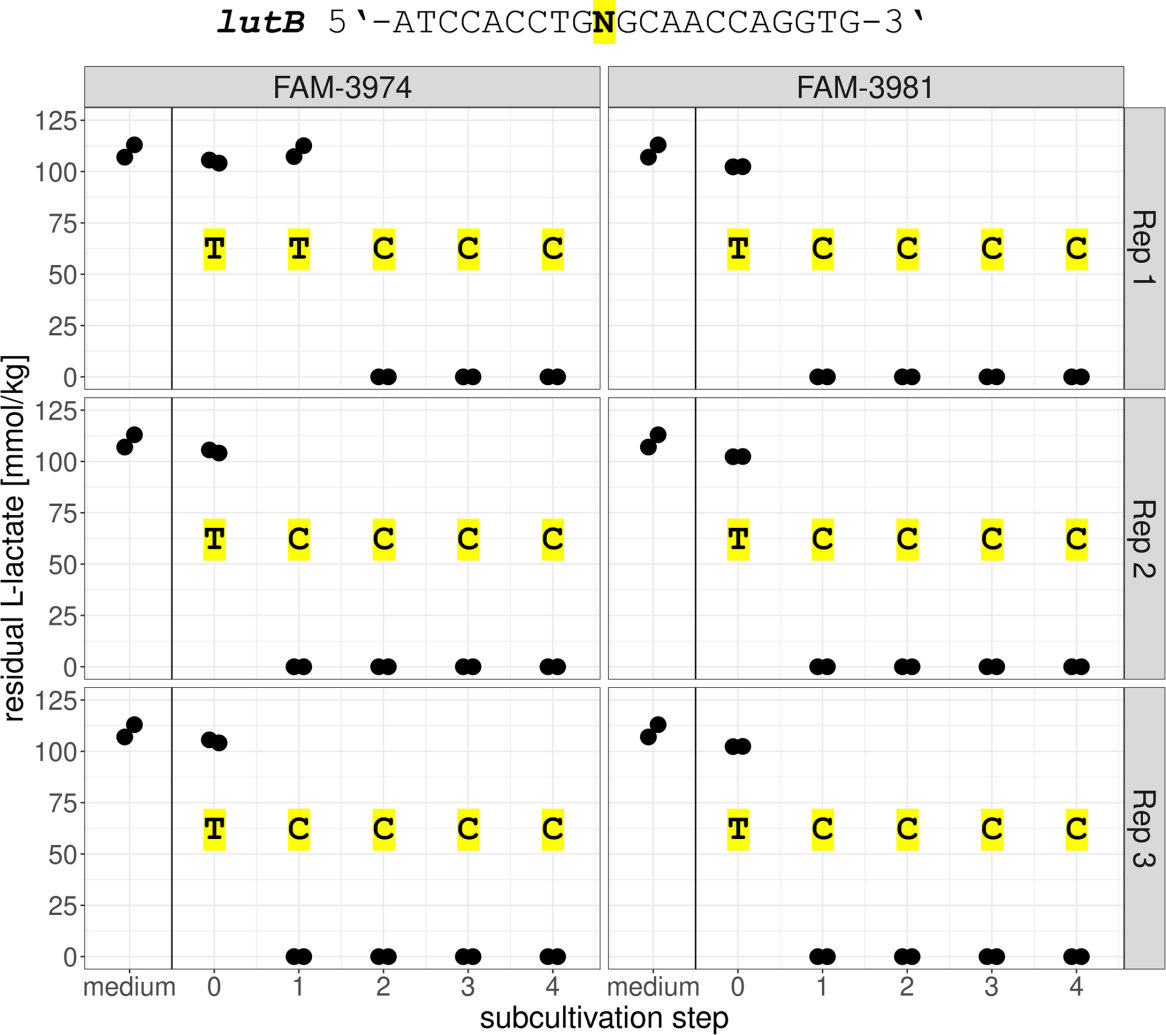
Main ALE experiment: lactate utilization assay of evolved isolates. Each dot represents one biological replicate. The nucleotide at position 1249 of the *lutB* gene is shown for each isolate on a yellow background.

To compare genotypes before and after a gain-of-function for L-lactate utilization, an ALE experiment was performed on L-lactate-negative strains. For each subcultivation step and biological replicate of the ALE experiment, a single colony was subcultured in MRS lactose broth and tested for L-lactate utilization. Figure 4 shows the residual L-lactate present in the cultivation medium after incubation of the isolates. The two original strains FAM-3974 and FAM-3981, that is, not subjected to ALE, were not able to utilize L-lactate under the assay’s conditions (subcultivation step 0). All isolates deriving from ALE completely utilized L-lactate, apart from FAM-3974_S1R1 (subcultivation step 1, replicate 1), which maintained the L-lactate-negative phenotype.

**Fig 5:**
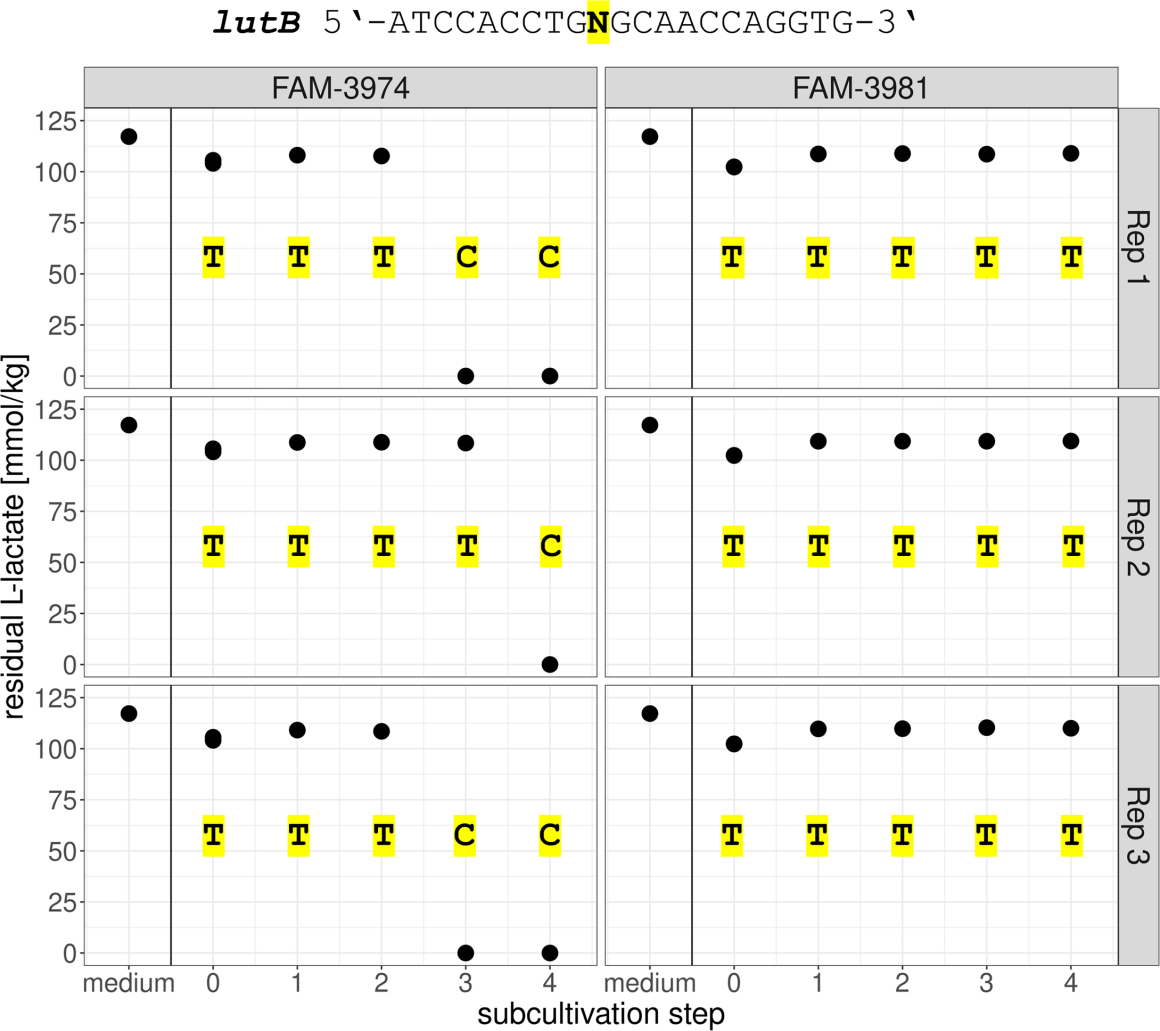
ALE control with no added lactate: lactate utilization assay of evolved isolates. Each dot represents one biological replicate. The nucleotide at position 1249 of the *lutB* gene is shown for each isolate on a yellow background.

As a control, we cultivated the two strains in YE broth (YEL-L broth with no added L-lactate). For one strain, namely FAM-3974, revertants occurred, but much later, after 3–4 weeks of cultivation, whereas in the case of FAM-3981, the ability to utilize L-lactate could not be restored in the time frame of the experiment, as shown in Fig. 5, highlighting the need for a selective pressure to recover the wild-type genotype.

After DNA extraction, the *lutB* gene of all ALE-derived isolates was amplified by PCR and Sanger-sequenced. As shown in Fig. 4, the nucleotide in position 1249 of the *lutB* gene was found to be thymine (T) in L-lactate-negative isolates and cytosine (C) in isolates able to utilize L-lactate. This polymorphism is responsible for an amino acid substitution in the predicted protein sequence, namely from cysteine (TGC codon) in L-lactate-negative isolates to arginine (CGC codon) in L-lactate-positive isolates.

As for the control experiment with no added lactate, we could observe the same correlation pattern between lactate utilization and the presence of the wild-type SNV in FAM-3974, while FAM-3981 maintained the L-lactate-negative genotype and phenotype throughout the experiment (Fig. 5).

## DISCUSSION

Two *P. freudenreichii* strains unable to degrade L-lactate were tested in a cheesemaking trial using the two isolates as ripening cultures, to evaluate the stability of the L-lactate-negative phenotype in a typical application of *P. freudenreichii*. After acidification of the curd, a roughly 1:1 mixture of D- and L-lactate is present (6). As expected, we could observe stable levels of L-lactate in the cheese over the 240 days of ripening, whereas D-lactate was completely depleted.

**Fig 6:**
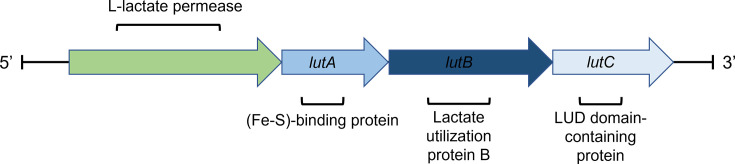
Structure of the *lutABC* locus in *P. freudenreichii* FAM-14222.

To link the L-lactate-negative phenotype to a genotype, the genomes of the two strains were sequenced. SNV calling highlighted the presence, among others, of a variant at position 1249 of the *lutB* gene (locus tag FAM14222-p2-1.1_001787), encoding the lactate utilization protein B, a putative iron-sulfur oxidoreductase (26), in both strains. The *lutB* gene is part of the *lutABC* operon, a highly conserved gene cluster previously described in *Shewanella oneidensis* (27) and *Bacillus subtilis* (28), and linked to L-lactate catabolism in these two species. Figure 6 shows the *lutABC* locus as annotated by the NCBI Prokaryotic Genome Annotation Pipeline, with an upstream L-lactate permease, as observed in many other species (28), which further suggests the potential relevance of this operon in L-lactate catabolism. The *lutABC* gene cluster was predicted by Operon-mapper (29) to be organized in an operon in *P. freudenreichii* FAM-14222, together with the L-lactate permease. The *lutB and lutC* proteins were found to contain the LUD domain, which was associated with lactate utilization in *Deinococcus radiodurans* and is present in high frequency among human gut microbes, where it may play a role in anaerobic metabolism (26).

**Fig 7:**
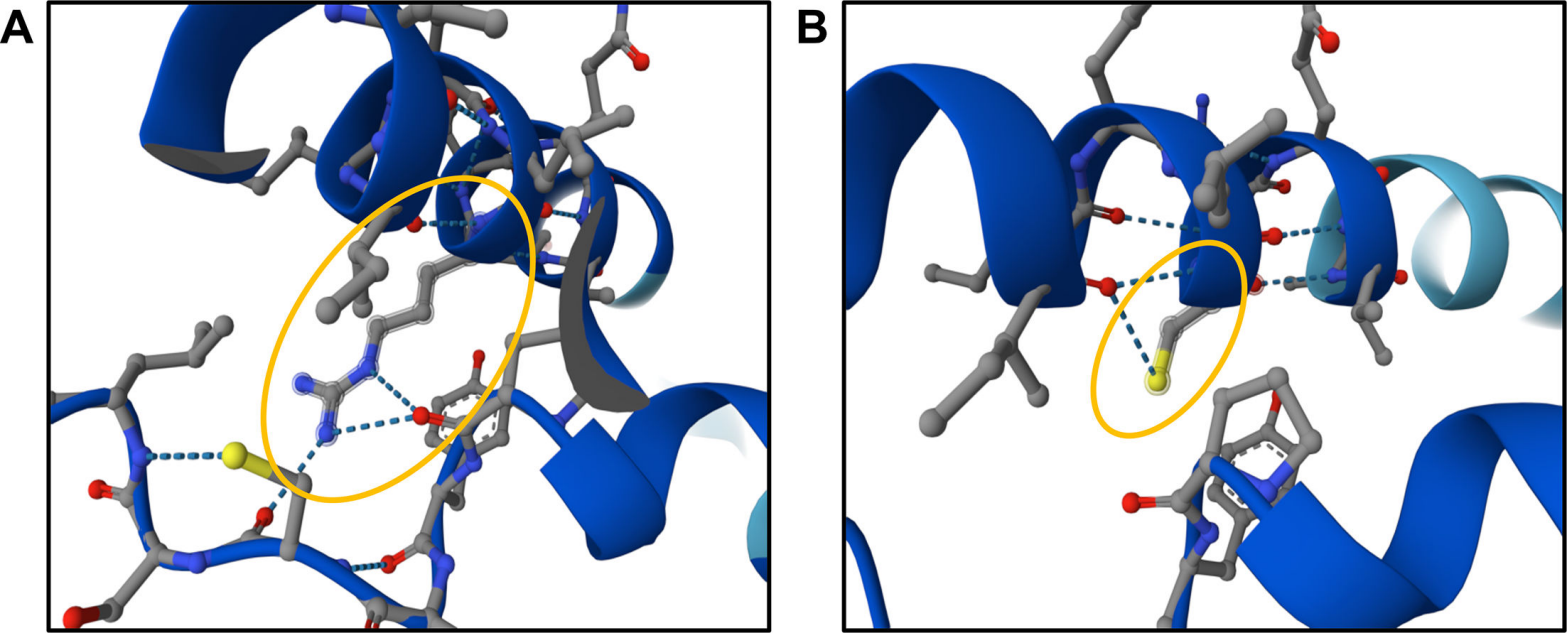
(A) Native structure of the *lutB* protein (sequence from FAM-14222), as predicted by Alphafold 3. Hydrogen bonds formed by the guanidino group of arginine 417 and the amino acid backbone of a neighboring loop are highlighted in the orange ellipse. (B) Mutated structure of the *lutB* protein (sequence from FAM-3974 and FAM-3981), as predicted by Alphafold 3. The side chain from cysteine 417 forms no hydrogen bonds with the neighboring loop, as highlighted in the orange ellipse.

Here, we found a correlation between the presence of an SNV in the *lutB* gene and the ability of *P. freudenreichii* isolates to catabolize L-lactate. At position 1249 of the *lutB* gene, cytosine is commonly found in wild-type strains, that is, in strains able to catabolize L-lactate. A BLASTn search against the nt/nr and wgs databases on NCBI with subsequent alignment of the *lutB* sequence from the two L-lactate-negative strains showed the uniqueness of thymine at this position (Fig. S1 and S2), as found in the two isolates object of this study, namely FAM-3974 and FAM-3981. This nucleotide substitution is predicted to cause an amino acid substitution from arginine (CGC codon) to cysteine (TGC codon) at position 417 in the protein sequence. These two amino acids differ drastically in their physicochemical properties and show dissimilar propensities for the formation of both α-helix and β-strand structures (30), which suggests a potential effect of this amino acid substitution on the protein’s secondary structure, and ultimately on its functionality. The 3D structure of the *lutB* protein for the two variants was predicted with AlphaFold 3 (31) running on the AlphaFold Server. In the case of the L-lactate-positive phenotype, we observed the presence of hydrogen bonds between the guanidino group of arginine 417 and the oxygen present in the side chains of amino acids in a neighboring loop, namely cysteine 393 and proline 390 (Fig. 7A). In the mutant structure present in the two L-lactate-negative strains, cysteine 417 lacks these hydrogen bonds, which may destabilize the tertiary structure of the protein (Fig. 7B). While AlphaFold did not predict any major changes in the secondary structure of the protein, it should be noted that this tool “has not been validated for predicting the effect of mutations,” as stated on AlphaFold’s website (https://alphafold.ebi.ac.uk/, accessed on 6 May 2025).

In our ALE experiment, the presence of thymine at position 1249 of the *lutB* gene was always associated with the isolates’ inability to catabolize L-lactate. This was the case for the two original strains FAM-3974 and FAM-3981 before the start of evolution, as well as for one isolate deriving from FAM-3974, in which thymine had not reverted to cysteine yet, as shown in Fig. 4 (FAM-3974, subcultivation step 1, replicate 1). In all other cases, the SNV reverted to wild type already during the first week of ALE and was consistently maintained for the whole duration of the experiment. All reverted isolates regained the ability to metabolize L-lactate. In a control experiment without added lactate, no revertant was observed for one strain, while for the other, they appeared much later than in the evolution experiment performed under conditions where lactate utilization would confer a competitive advantage. In these revertants too, L-lactate utilization correlated with the occurrence of a cytosine at position 1249. We speculate that L-lactate production during the initial phases of growth (32) may have given a competitive advantage to cells able to re-metabolize it.

Interestingly, no variants were detected in the two annotated L-lactate dehydrogenases, nor in the D-lactate dehydrogenase, which are thus identical to their homologs in FAM-14222. This observation further supports the hypothesis that functional lactate dehydrogenases are not sufficient for *P. freudenreichii* to utilize L-lactate. Moreover, the expression of the *lutABC* operon was found to be upregulated during L-lactate consumption and downregulated in the stationary phase, as reported by a recent transcriptomics study on *P. freudenreichii* (33). It is interesting to note that, in contrast to ALE, the L-lactate-negative phenotype of the two strains was stable during cheese ripening. The two conditions differ radically from each other, as ALE allows highly controlled cultivation parameters that were optimized for providing revertants with a competitive advantage, while cheesemaking conditions are much more complex and involve, among other parameters, co-culturing with lactic acid starters. We speculate that, although revertants able to utilize L-lactate may naturally arise during cheese maturation, being embedded in a solid matrix might prevent these cells from colonizing the entire bulk of the cheese, as opposed to ALE, where a liquid medium was used. Moreover, after D-lactate is depleted in the cheese, other factors, such as molecules used for inter-cellular communication, might be suppressing growth, even in the presence of remaining levels of L-lactate. The commercial culture used as a control metabolized both lactate enantiomers, with a preference for L-lactate, as previously reported for wild-type *P. freudenreichii* strains (10, 12). This observation highlights the potential of FAM-3974 and FAM-3981 as ripening cultures for Swiss-type cheese. Since only about half of the total lactate present in the cheese could be metabolized, we expect the two strains to show diminished levels of CO_2_ production compared to wild types, which may be a valuable property for the reduction of cheese defects such as cracks and late fermentation, without the addition of facultatively heterofermentative Lactobacilli as starter LAB, which are associated with poorer sensory properties of the cheese (7). Moreover, the use of L-lactate-negative strains led to D-lactate-free cheese at the end of maturation, which is regarded as a desirable feature due to unfavorable implications of D-lactate consumption (34).

### Conclusions

Our findings support the involvement of the *lutABC* operon in L-lactate catabolism also in *P. freudenreichii*, as previously hypothesized by McCubbin et al. (13). Furthermore, we report the essential role of the arginine located in position 417 in the functionality of the protein. The use of ALE combined with whole-genome sequencing as a general discovery method is on the rise (35). However, among several mutations found in the L-lactate-negative strains compared to a wild type, only the *lutB* gene was characterized in the evolved isolates, due to its previous annotation and the high plausibility of being involved in L-lactate catabolism. While the evidence for the role of *lutB* is strong, a limitation of the present approach lies in excluding the potential role of other genes that were not sequenced after evolution. Whole-genome sequencing of evolved isolates may deliver additional insights in this regard. Whether other genes in the operon are also essential remains to be determined, for example, through a targeted mutagenesis of each gene. Moreover, knock-out/complementation experiments of *lutB* may provide additional confirmation of the essential role of this gene in L-lactate catabolism. Finally, the fact that only L-lactate, but not D-lactate metabolism, was affected by the amino acid substitution in *lutB* suggests the existence of alternative catabolic pathways beyond the *lutABC* operon yet to be characterized. Further research is required for the elucidation of the genetic background of D-lactate utilization in *P. freudenreichii*.

As for potential applications of the two strains of this study, the L-lactate-negative phenotype may be of interest for culture development for Swiss-type cheese. Compared to wild-type *P. freudenreichii*, only half of the substrate present in the curd, that is, only D-lactate, is available for the growth of L-lactate-negative strains. Accordingly, reduced production of end metabolites is expected, including CO_2_. This property may contribute to the reduction of cheese defects during ripening.

## Data Availability

Raw reads for FAM-3974 and FAM-3981 are available in NCBI under BioProject no. PRJNA1234514.

## SUPPLEMENTAL MATERIAL

Figure S1 aem.00599-25-s0001.pdf BLASTn search against the nt/nr database on NCBI, showing the uniqueness of thymine instead of cytosine at position 1249 of the *lutB* gene in *P. freudenreichii*.

Figure S2 aem.00599-25-s0002.pdf BLASTn search against the wgs database on NCBI, showing the uniqueness of thymine instead of cytosine at position 1249 of the *lutB* gene in *P. freudenreichii*.

Supplemental legends aem.00599-25-s0003.docx Legends for Table S1 and Fig. S1 and S2.

Table S1 aem.00599-25-s0004.html Overview of all detected non-silent variants in FAM-3974 and FAM-3981, as annotated by SnpEff.

ASM does not own the copyrights to Supplemental Material that may be linked to, or accessed through, an article. The authors have granted ASM a non-exclusive, world-wide license to publish the Supplemental Material files. Please contact the corresponding author directly for reuse.
