# Supplementary material for: A single-nucleotide variant conditions the ability vs. inability of Propionibacterium freudenreichii to utilize L-lactate
Source: Appl Environ Microbiol. 2025 Jun 12;91(7):e00599-25. doi: 10.1128/aem.00599-25 (PMC12285252; doi:10.1128/aem.00599-25)
Supplement: Figure S1 — BLASTn search against the nt/nr database on NCBI, showing the uniqueness of thymine instead of cytosine at position 1249 of the lutB gene in P. freudenreichii. [file aem.00599-25-s0001.pdf]

NCBI Multiple Sequence Alignment Viewer, Version 1.25.3

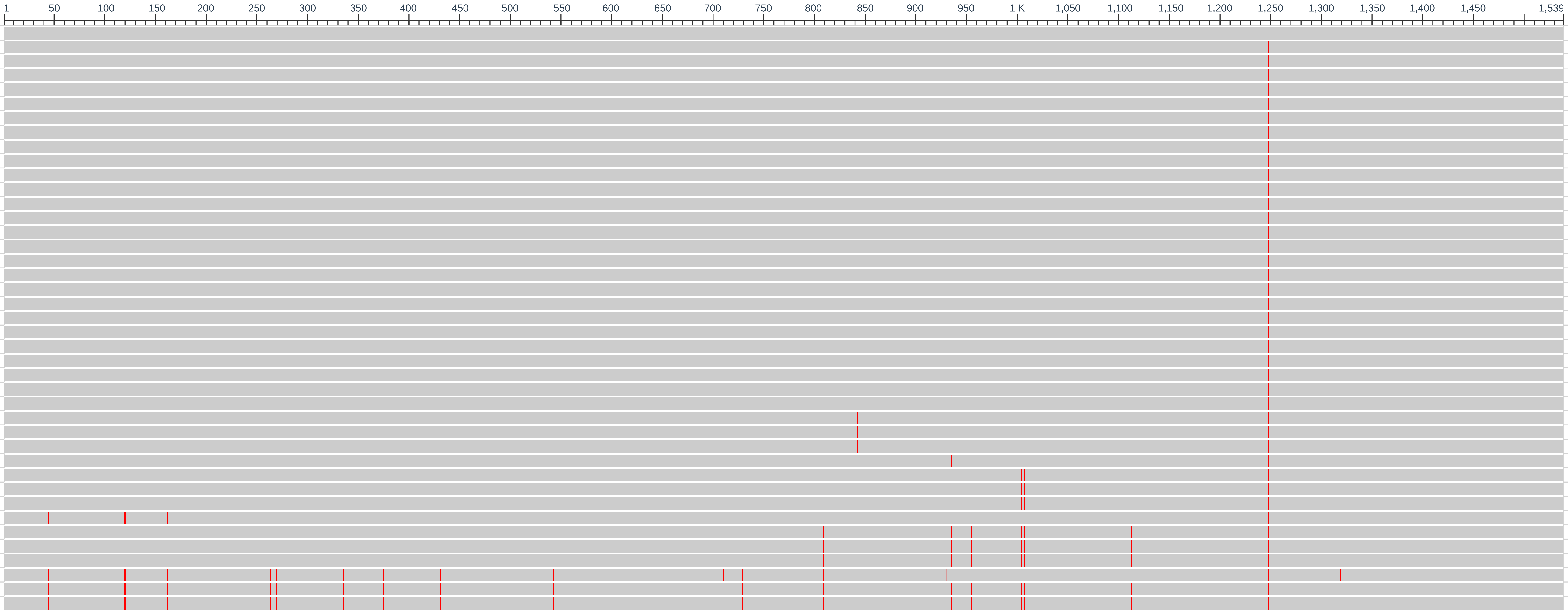

Sequence ID		Start	End	Organism
Query_6267493	(+)	1	1,539	
CP053853.1	(+)	1,001,...	1,003,...	Propionibacterium freud...
LT618793.1	(+)	519,030	520,568	Propionibacterium freud...
LT618792.1	(+)	400,266	401,804	Propionibacterium freud...
LT618791.1	(+)	400,266	401,804	Propionibacterium freud...
LT618786.1	(+)	594,452	595,990	Propionibacterium freud...
LT618785.1	(+)	595,909	597,447	Propionibacterium freud...
LT618783.1	(+)	432,389	433,927	Propionibacterium freud...
LT618782.1	(+)	576,214	577,752	Propionibacterium freud...
LT618781.1	(+)	577,657	579,195	Propionibacterium freud...
LT618780.1	(+)	547,630	549,168	Propionibacterium freud...
LT618779.1	(+)	564,350	565,888	Propionibacterium freud...
LT618777.1	(+)	575,476	577,014	Propionibacterium freud...
LT618776.1	(+)	575,476	577,014	Propionibacterium freud...
LT599498.1	(+)	605,284	606,822	Propionibacterium freud...
LT593929.1	(+)	553,618	555,156	Propionibacterium freud...
LT576042.1	(+)	584,448	585,986	Propionibacterium freud...
LT576038.1	(+)	564,380	565,918	Propionibacterium freud...
LT576033.1	(-)	2,034,...	2,033,...	Propionibacterium freud...
LT576032.1	(+)	571,059	572,597	Propionibacterium freud...
LR698993.1	(+)	520,715	522,253	Propionibacterium freud...
LN997841.1	(-)	1,905,...	1,904,...	Propionibacterium freud...
CP124208.1	(+)	452,318	453,856	Propionibacterium freud...
CP120880.1	(-)	2,100,...	2,099,...	Propionibacterium freud...
CP120879.1	(-)	2,099,...	2,097,...	Propionibacterium freud...
CP085641.1	(+)	214,127	215,665	Propionibacterium freud...
CP085639.1	(-)	2,062,...	2,061,...	Propionibacterium freud...
CP030279.1	(+)	600,372	601,910	Propionibacterium freud...
LT618790.1	(+)	602,347	603,885	Propionibacterium freud...
LT618789.1	(+)	602,347	603,885	Propionibacterium freud...
LM676443.1	(+)	82,997	84,535	Propionibacterium freud...
LT604998.1	(+)	552,889	554,427	Propionibacterium freud...
LT576787.1	(+)	552,889	554,427	Propionibacterium freud...
CP116039.1	(-)	2,032,...	2,031,...	Propionibacterium freud...
CP010341.1	(-)	1,993,...	1,991,...	Propionibacterium freud...
LT618788.1	(-)	2,003,...	2,002,...	Propionibacterium freud...
LT618787.1	(-)	2,007,...	2,005,...	Propionibacterium freud...
FN806773.1	(-)	2,003,...	2,001,...	Propionibacterium freud...
CP085640.1	(-)	2,315,...	2,314,...	Propionibacterium freud...
LT576034.1	(+)	563,776	565,314	Propionibacterium freud...
CP120878.1	(-)	2,133,...	2,131,...	Propionibacterium freud...
