# Supplementary figures and images for: A single-nucleotide variant conditions the ability vs. inability of Propionibacterium freudenreichii to utilize L-lactate
Source: Appl Environ Microbiol. 2025 Jun 12;91(7):e00599-25. doi: 10.1128/aem.00599-25 (PMC12285252; doi:10.1128/aem.00599-25)

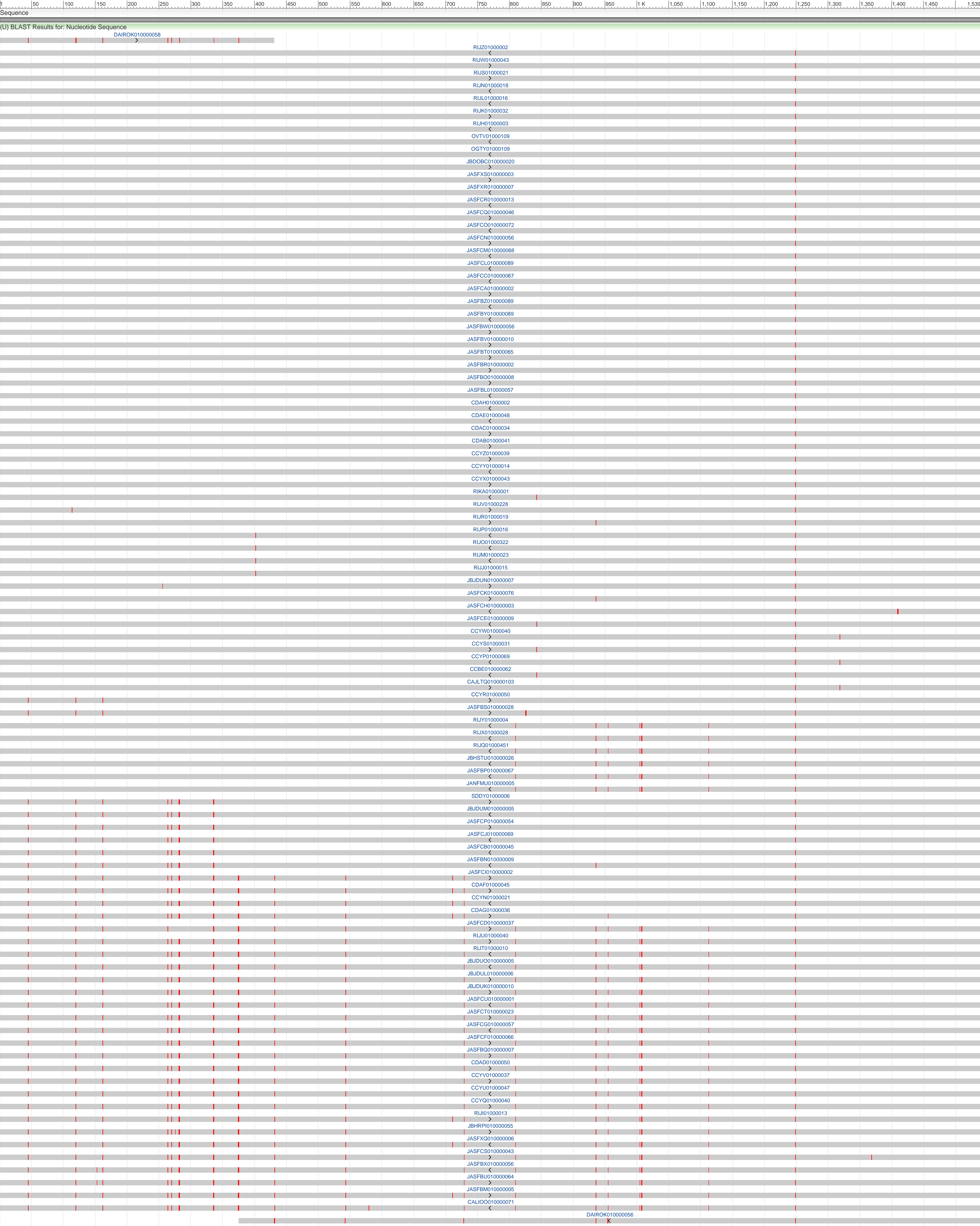

Supplement: Figure S2 — BLASTn search against the wgs database on NCBI, showing the uniqueness of thymine instead of cytosine at position 1249 of the lutB gene in P. freudenreichii. [file aem.00599-25-s0002.pdf]
