# Supplementary material for: A single-nucleotide variant conditions the ability vs. inability of Propionibacterium freudenreichii to utilize L-lactate
Source: Appl Environ Microbiol. 2025 Jun 12;91(7):e00599-25. doi: 10.1128/aem.00599-25 (PMC12285252; doi:10.1128/aem.00599-25)
Supplement: Supplemental legends — Legends for Table S1 and Fig. S1 and S2. [file aem.00599-25-s0003.docx]

Table S1: Overview of all detected non-silent variants in FAM-3974 and FAM-3981, as annotated by SnpEff.

Figure S1: BLASTn search against the nt/nr database on NCBI, showing the uniqueness of thymine instead of cytosine at position 1249 of the *lutB* gene in *P. freudenreichii*.

Figure S2: BLASTn search against the wgs database on NCBI, showing the uniqueness of thymine instead of cytosine at position 1249 of the *lutB* gene in *P. freudenreichii*.
