# Supplementary material for: A single-nucleotide variant conditions the ability vs. inability of Propionibacterium freudenreichii to utilize L-lactate
Source: Appl Environ Microbiol. 2025 Jun 12;91(7):e00599-25. doi: 10.1128/aem.00599-25 (PMC12285252; doi:10.1128/aem.00599-25)
Supplement: Table S1 — Overview of all detected non-silent variants in FAM-3974 and FAM-3981, as annotated by SnpEff. [file aem.00599-25-s0004.html]

|  | Pos | Type | X. | Y | Strain | Reference | FAM14222.genome..bp. | snpEff | annotation | locus\_tag |
| --- | --- | --- | --- | --- | --- | --- | --- | --- | --- | --- |
| 1 | 14103 | SNV | 0.53 | 1.00 | FAM-3974 | FAM14222 | 2655381 | MODERATE | HAMP domain-containing sensor histidine kinase | FAM14222p2\_000018 |
| 2 | 36506 | SNV | 1.37 | 1.00 | FAM-3974 | FAM14222 |  | LOW | sugar phosphate isomerase/epimerase | FAM14222p2\_000034 |
| 3 | 42375 | SNV | 1.60 | 1.00 | FAM-3974 | FAM14222 |  | MODERATE | glycerophosphodiester phosphodiesterase family protein | FAM14222p2\_000042 |
| 4 | 51224 | SNV | 1.93 | 1.00 | FAM-3974 | FAM14222 |  | LOW | glutamate decarboxylase | FAM14222p2\_000049 |
| 5 | 116438 | SNV | 4.38 | 1.00 | FAM-3974 | FAM14222 |  | LOW | hypothetical protein | FAM14222p2\_000105 |
| 6 | 150602 | SNV | 5.67 | 1.00 | FAM-3974 | FAM14222 |  | MODERATE | IS481-like element ISPfr5 family transposase | FAM14222p2\_000131 |
| 7 | 180639 | SNV | 6.80 | 1.00 | FAM-3974 | FAM14222 |  | LOW | DNA gyrase subunit A | FAM14222p2\_000160 |
| 8 | 192771 | INDEL | 7.26 | 1.10 | FAM-3974 | FAM14222 |  | HIGH | DUF262 domain-containing protein | FAM14222p2\_000167 |
| 9 | 206793 | SNV | 7.79 | 1.00 | FAM-3974 | FAM14222 |  | MODERATE | MFS transporter | FAM14222p2\_000179 |
| 10 | 218994 | SNV | 8.25 | 1.00 | FAM-3974 | FAM14222 |  | MODERATE | amino acid permease | FAM14222p2\_000188 |
| 11 | 219226 | SNV | 8.26 | 1.00 | FAM-3974 | FAM14222 |  | LOW | amino acid permease | FAM14222p2\_000188 |
| 12 | 219463 | SNV | 8.26 | 1.00 | FAM-3974 | FAM14222 |  | LOW | amino acid permease | FAM14222p2\_000188 |
| 13 | 221986 | SNV | 8.36 | 1.00 | FAM-3974 | FAM14222 |  | MODERATE | IMPACT family protein | FAM14222p2\_000191 |
| 14 | 246817 | SNV | 9.29 | 1.00 | FAM-3974 | FAM14222 |  | MODERATE | ABC transporter permease | FAM14222p2\_000208 |
| 15 | 324128 | SNV | 12.21 | 1.00 | FAM-3974 | FAM14222 |  | MODERATE | FHA domain-containing protein | FAM14222p2\_000264 |
| 16 | 447607 | SNV | 16.86 | 1.00 | FAM-3974 | FAM14222 |  | n.a. | ID=rna-FAM14222p2\_000362;Parent=gene-FAM14222p2\_000362;anticodon=(pos:complement(447641..447643));gbkey=tRNA;inference=COORDINATES: profile:tRNAscan-SE:2.0.12;locus\_tag=FAM14222p2\_000362;product=tRNA-Leu; ID=exon-FAM14222p2\_000362-1;Parent=rna-FAM14222p2\_000362;anticodon=(pos:complement(447641..447643));gbkey=tRNA;inference=COORDINATES: profile:tRNAscan-SE:2.0.12;locus\_tag=FAM14222p2\_000362;product=tRNA-Leu | FAM14222p2\_000362; FAM14222p2\_000362 |
| 17 | 518797 | SNV | 19.54 | 1.00 | FAM-3974 | FAM14222 |  | LOW | DUF3073 domain-containing protein | FAM14222p2\_000419 |
| 18 | 520441 | SNV | 19.60 | 1.00 | FAM-3974 | FAM14222 |  | n.a. | no hit | no hit |
| 19 | 542784 | INDEL | 20.44 | 1.10 | FAM-3974 | FAM14222 |  | HIGH | TM0106 family RecB-like putative nuclease | FAM14222p2\_000446 |
| 20 | 548547 | SNV | 20.66 | 1.00 | FAM-3974 | FAM14222 |  | MODERATE | chorismate-binding protein | FAM14222p2\_000450 |
| 21 | 700169 | INDEL | 26.37 | 1.10 | FAM-3974 | FAM14222 |  | n.a. | no hit | no hit |
| 22 | 717003 | SNV | 27.00 | 1.00 | FAM-3974 | FAM14222 |  | MODERATE | iron chelate uptake ABC transporter family permease subunit | FAM14222p2\_000617 |
| 23 | 771619 | SNV | 29.06 | 1.00 | FAM-3974 | FAM14222 |  | LOW | Type 1 glutamine amidotransferase-like domain-containing protein | FAM14222p2\_000667 |
| 24 | 780356 | SNV | 29.39 | 1.00 | FAM-3974 | FAM14222 |  | LOW | hypothetical protein | FAM14222p2\_000674 |
| 25 | 881076 | INDEL | 33.18 | 1.10 | FAM-3974 | FAM14222 |  | HIGH | dihydroxyacetone kinase subunit DhaL | FAM14222p2\_000767 |
| 26 | 881077 | INDEL | 33.18 | 1.10 | FAM-3974 | FAM14222 |  | MODERATE | dihydroxyacetone kinase subunit DhaL | FAM14222p2\_000767 |
| 27 | 881078 | INDEL | 33.18 | 1.10 | FAM-3974 | FAM14222 |  | HIGH | dihydroxyacetone kinase subunit DhaL | FAM14222p2\_000767 |
| 28 | 881079 | INDEL | 33.18 | 1.10 | FAM-3974 | FAM14222 |  | MODERATE | dihydroxyacetone kinase subunit DhaL | FAM14222p2\_000767 |
| 29 | 906325 | SNV | 34.13 | 1.00 | FAM-3974 | FAM14222 |  | MODERATE | hypothetical protein | FAM14222p2\_000784 |
| 30 | 977165 | SNV | 36.80 | 1.00 | FAM-3974 | FAM14222 |  | LOW | PhoH family protein | FAM14222p2\_000848 |
| 31 | 1030213 | SNV | 38.80 | 1.00 | FAM-3974 | FAM14222 |  | LOW | VWA domain-containing protein | FAM14222p2\_000903 |
| 32 | 1046517 | INDEL | 39.41 | 1.10 | FAM-3974 | FAM14222 |  | n.a. | no hit | no hit |
| 33 | 1151190 | SNV | 43.35 | 1.00 | FAM-3974 | FAM14222 |  | n.a. | no hit | no hit |
| 34 | 1249699 | SNV | 47.06 | 1.00 | FAM-3974 | FAM14222 |  | MODERATE | methionyl-tRNA formyltransferase | FAM14222p2\_001100 |
| 35 | 1325286 | SNV | 49.91 | 1.00 | FAM-3974 | FAM14222 |  | MODERATE | L-threonylcarbamoyladenylate synthase | FAM14222p2\_001162 |
| 36 | 1376628 | SNV | 51.84 | 1.00 | FAM-3974 | FAM14222 |  | MODERATE | aconitate hydratase AcnA | FAM14222p2\_001216 |
| 37 | 1386059 | SNV | 52.20 | 1.00 | FAM-3974 | FAM14222 |  | MODERATE | TRAM domain-containing protein | FAM14222p2\_001224 |
| 38 | 1465456 | INDEL | 55.19 | 1.10 | FAM-3974 | FAM14222 |  | HIGH | citramalate synthase | FAM14222p2\_001286 |
| 39 | 1528357 | SNV | 57.56 | 1.00 | FAM-3974 | FAM14222 |  | MODERATE | ATP-dependent zinc metalloprotease FtsH | FAM14222p2\_001346 |
| 40 | 1555515 | SNV | 58.58 | 1.00 | FAM-3974 | FAM14222 |  | MODERATE | chromosome segregation protein SMC | FAM14222p2\_001376 |
| 41 | 1555894 | SNV | 58.59 | 1.00 | FAM-3974 | FAM14222 |  | MODERATE | chromosome segregation protein SMC | FAM14222p2\_001376 |
| 42 | 1561287 | INDEL | 58.80 | 1.10 | FAM-3974 | FAM14222 |  | n.a. | no hit | no hit |
| 43 | 1563365 | INDEL | 58.88 | 1.10 | FAM-3974 | FAM14222 |  | HIGH | pyridoxamine 5'-phosphate oxidase family protein | FAM14222p2\_001384 |
| 44 | 1563448 | INDEL | 58.88 | 1.10 | FAM-3974 | FAM14222 |  | HIGH | pyridoxamine 5'-phosphate oxidase family protein | FAM14222p2\_001384 |
| 45 | 1563458 | INDEL | 58.88 | 1.10 | FAM-3974 | FAM14222 |  | HIGH | pyridoxamine 5'-phosphate oxidase family protein | FAM14222p2\_001384 |
| 46 | 1563690 | INDEL | 58.89 | 1.10 | FAM-3974 | FAM14222 |  | HIGH | hypothetical protein | FAM14222p2\_001385 |
| 47 | 1563764 | INDEL | 58.89 | 1.10 | FAM-3974 | FAM14222 |  | MODERATE | hypothetical protein | FAM14222p2\_001385 |
| 48 | 1563767 | INDEL | 58.89 | 1.10 | FAM-3974 | FAM14222 |  | HIGH | hypothetical protein | FAM14222p2\_001385 |
| 49 | 1563769 | INDEL | 58.89 | 1.10 | FAM-3974 | FAM14222 |  | HIGH | hypothetical protein | FAM14222p2\_001385 |
| 50 | 1563770 | INDEL | 58.89 | 1.10 | FAM-3974 | FAM14222 |  | HIGH | hypothetical protein | FAM14222p2\_001385 |
| 51 | 1563853 | INDEL | 58.89 | 1.10 | FAM-3974 | FAM14222 |  | HIGH | ATP-dependent DNA helicase RecG | FAM14222p2\_001386 |
| 52 | 1583641 | SNV | 59.64 | 1.00 | FAM-3974 | FAM14222 |  | n.a. | no hit | no hit |
| 53 | 1676006 | SNV | 63.12 | 1.00 | FAM-3974 | FAM14222 |  | MODERATE | UDP-N-acetylmuramoyl-L-alanyl-D-glutamate--2%2C6-diaminopimelate ligase | FAM14222p2\_001489 |
| 54 | 1697144 | INDEL | 63.91 | 1.10 | FAM-3974 | FAM14222 |  | n.a. | 1-acyl-sn-glycerol-3-phosphate acyltransferase | FAM14222p2\_001506 |
| 55 | 1729970 | SNV | 65.15 | 1.00 | FAM-3974 | FAM14222 |  | LOW | DUF2510 domain-containing protein | FAM14222p2\_001537 |
| 56 | 1786392 | SNV | 67.27 | 1.00 | FAM-3974 | FAM14222 |  | MODERATE | thiazole synthase | FAM14222p2\_001591 |
| 57 | 1791074 | INDEL | 67.45 | 1.10 | FAM-3974 | FAM14222 |  | n.a. | no hit | no hit |
| 58 | 1829437 | SNV | 68.90 | 1.00 | FAM-3974 | FAM14222 |  | LOW | AI-2E family transporter | FAM14222p2\_001633 |
| 59 | 1859420 | SNV | 70.02 | 1.00 | FAM-3974 | FAM14222 |  | MODERATE | transcription-repair coupling factor | FAM14222p2\_001664 |
| 60 | 1916193 | SNV | 72.16 | 1.00 | FAM-3974 | FAM14222 |  | MODERATE | conjugal transfer protein TrbL | FAM14222p2\_001719 |
| 61 | 1944789 | SNV | 73.24 | 1.00 | FAM-3974 | FAM14222 |  | LOW | NCS2 family permease | FAM14222p2\_001755 |
| 62 | 1953368 | SNV | 73.56 | 1.00 | FAM-3974 | FAM14222 |  | n.a. | no hit | no hit |
| 63 | 1974195 | INDEL | 74.35 | 1.10 | FAM-3974 | FAM14222 |  | HIGH | NAD-binding protein | FAM14222p2\_001776 |
| 64 | 1976156 | SNV | 74.42 | 1.00 | FAM-3974 | FAM14222 |  | MODERATE | DUF6394 family protein | FAM14222p2\_001777 |
| 65 | 1977768 | SNV | 74.48 | 1.00 | FAM-3974 | FAM14222 |  | LOW | VTT domain-containing protein | FAM14222p2\_001779 |
| 66 | 1984893 | SNV | 74.75 | 1.00 | FAM-3974 | FAM14222 |  | MODERATE | DUF4032 domain-containing protein | FAM14222p2\_001784 |
| 67 | 1986985 | SNV | 74.83 | 1.00 | FAM-3974 | FAM14222 |  | MODERATE | lactate utilization protein B | FAM14222p2\_001787 |
| 68 | 2006343 | SNV | 75.56 | 1.00 | FAM-3974 | FAM14222 |  | LOW | phosphoglyceromutase | FAM14222p2\_001803 |
| 69 | 2012668 | SNV | 75.80 | 1.00 | FAM-3974 | FAM14222 |  | n.a. | no hit | no hit |
| 70 | 2018373 | SNV | 76.01 | 1.00 | FAM-3974 | FAM14222 |  | LOW | ferrochelatase | FAM14222p2\_001814 |
| 71 | 2046638 | SNV | 77.08 | 1.00 | FAM-3974 | FAM14222 |  | MODERATE | zinc-binding dehydrogenase | FAM14222p2\_001836 |
| 72 | 2065575 | SNV | 77.79 | 1.00 | FAM-3974 | FAM14222 |  | n.a. | no hit | no hit |
| 73 | 2086097 | SNV | 78.56 | 1.00 | FAM-3974 | FAM14222 |  | MODERATE | copper-translocating P-type ATPase | FAM14222p2\_001871 |
| 74 | 2122055 | SNV | 79.92 | 1.00 | FAM-3974 | FAM14222 |  | n.a. | no hit | no hit |
| 75 | 2180719 | SNV | 82.12 | 1.00 | FAM-3974 | FAM14222 |  | LOW | D-alanyl-D-alanine carboxypeptidase/D-alanyl-D-alanine-endopeptidase | FAM14222p2\_001957 |
| 76 | 2183926 | SNV | 82.25 | 1.00 | FAM-3974 | FAM14222 |  | LOW | histidine phosphatase family protein | FAM14222p2\_001960 |
| 77 | 2196694 | SNV | 82.73 | 1.00 | FAM-3974 | FAM14222 |  | LOW | hypothetical protein | FAM14222p2\_001972 |
| 78 | 2206179 | SNV | 83.08 | 1.00 | FAM-3974 | FAM14222 |  | MODERATE | methyltransferase | FAM14222p2\_001977 |
| 79 | 2230703 | SNV | 84.01 | 1.00 | FAM-3974 | FAM14222 |  | n.a. | no hit | no hit |
| 80 | 2258759 | SNV | 85.06 | 1.00 | FAM-3974 | FAM14222 |  | MODERATE | hypothetical protein | FAM14222p2\_002027 |
| 81 | 2289984 | SNV | 86.24 | 1.00 | FAM-3974 | FAM14222 |  | LOW | bifunctional diaminohydroxyphosphoribosylaminopyrimidine deaminase/5-amino-6-(5-phosphoribosylamino)uracil reductase RibD | FAM14222p2\_002050 |
| 82 | 2309729 | SNV | 86.98 | 1.00 | FAM-3974 | FAM14222 |  | MODERATE | nitrite/sulfite reductase | FAM14222p2\_002067 |
| 83 | 2325602 | SNV | 87.58 | 1.00 | FAM-3974 | FAM14222 |  | LOW | molybdate ABC transporter substrate-binding protein | FAM14222p2\_002077 |
| 84 | 2339188 | SNV | 88.09 | 1.00 | FAM-3974 | FAM14222 |  | LOW | molybdenum cofactor biosynthesis protein MoaE | FAM14222p2\_002087 |
| 85 | 2394795 | SNV | 90.19 | 1.00 | FAM-3974 | FAM14222 |  | n.a. | no hit | no hit |
| 86 | 2408238 | INDEL | 90.69 | 1.10 | FAM-3974 | FAM14222 |  | n.a. | no hit | no hit |
| 87 | 2634219 | INDEL | 99.20 | 1.10 | FAM-3974 | FAM14222 |  | n.a. | no hit | no hit |
| 88 | 2634233 | INDEL | 99.20 | 1.10 | FAM-3974 | FAM14222 |  | n.a. | no hit | no hit |
| 89 | 2634240 | INDEL | 99.20 | 1.10 | FAM-3974 | FAM14222 |  | n.a. | no hit | no hit |
| 90 | 2634248 | INDEL | 99.20 | 1.10 | FAM-3974 | FAM14222 |  | n.a. | no hit | no hit |
| 91 | 2634254 | INDEL | 99.20 | 1.10 | FAM-3974 | FAM14222 |  | n.a. | no hit | no hit |
| 92 | 14103 | SNV | 0.53 | 1.00 | FAM-3981 | FAM14222 |  | MODERATE | HAMP domain-containing sensor histidine kinase | FAM14222p2\_000018 |
| 93 | 36506 | SNV | 1.37 | 1.00 | FAM-3981 | FAM14222 |  | LOW | sugar phosphate isomerase/epimerase | FAM14222p2\_000034 |
| 94 | 42375 | SNV | 1.60 | 1.00 | FAM-3981 | FAM14222 |  | MODERATE | glycerophosphodiester phosphodiesterase family protein | FAM14222p2\_000042 |
| 95 | 51224 | SNV | 1.93 | 1.00 | FAM-3981 | FAM14222 |  | LOW | glutamate decarboxylase | FAM14222p2\_000049 |
| 96 | 116438 | SNV | 4.38 | 1.00 | FAM-3981 | FAM14222 |  | LOW | hypothetical protein | FAM14222p2\_000105 |
| 97 | 150602 | SNV | 5.67 | 1.00 | FAM-3981 | FAM14222 |  | MODERATE | IS481-like element ISPfr5 family transposase | FAM14222p2\_000131 |
| 98 | 180639 | SNV | 6.80 | 1.00 | FAM-3981 | FAM14222 |  | LOW | DNA gyrase subunit A | FAM14222p2\_000160 |
| 99 | 192771 | INDEL | 7.26 | 1.10 | FAM-3981 | FAM14222 |  | HIGH | DUF262 domain-containing protein | FAM14222p2\_000167 |
| 100 | 206793 | SNV | 7.79 | 1.00 | FAM-3981 | FAM14222 |  | MODERATE | MFS transporter | FAM14222p2\_000179 |
| 101 | 218994 | SNV | 8.25 | 1.00 | FAM-3981 | FAM14222 |  | MODERATE | amino acid permease | FAM14222p2\_000188 |
| 102 | 219226 | SNV | 8.26 | 1.00 | FAM-3981 | FAM14222 |  | LOW | amino acid permease | FAM14222p2\_000188 |
| 103 | 219463 | SNV | 8.26 | 1.00 | FAM-3981 | FAM14222 |  | LOW | amino acid permease | FAM14222p2\_000188 |
| 104 | 221986 | SNV | 8.36 | 1.00 | FAM-3981 | FAM14222 |  | MODERATE | IMPACT family protein | FAM14222p2\_000191 |
| 105 | 246817 | SNV | 9.29 | 1.00 | FAM-3981 | FAM14222 |  | MODERATE | ABC transporter permease | FAM14222p2\_000208 |
| 106 | 324128 | SNV | 12.21 | 1.00 | FAM-3981 | FAM14222 |  | MODERATE | FHA domain-containing protein | FAM14222p2\_000264 |
| 107 | 447607 | SNV | 16.86 | 1.00 | FAM-3981 | FAM14222 |  | n.a. | ID=rna-FAM14222p2\_000362;Parent=gene-FAM14222p2\_000362;anticodon=(pos:complement(447641..447643));gbkey=tRNA;inference=COORDINATES: profile:tRNAscan-SE:2.0.12;locus\_tag=FAM14222p2\_000362;product=tRNA-Leu; ID=exon-FAM14222p2\_000362-1;Parent=rna-FAM14222p2\_000362;anticodon=(pos:complement(447641..447643));gbkey=tRNA;inference=COORDINATES: profile:tRNAscan-SE:2.0.12;locus\_tag=FAM14222p2\_000362;product=tRNA-Leu | FAM14222p2\_000362; FAM14222p2\_000362 |
| 108 | 518797 | SNV | 19.54 | 1.00 | FAM-3981 | FAM14222 |  | LOW | DUF3073 domain-containing protein | FAM14222p2\_000419 |
| 109 | 520441 | SNV | 19.60 | 1.00 | FAM-3981 | FAM14222 |  | n.a. | no hit | no hit |
| 110 | 542784 | INDEL | 20.44 | 1.10 | FAM-3981 | FAM14222 |  | HIGH | TM0106 family RecB-like putative nuclease | FAM14222p2\_000446 |
| 111 | 545176 | SNV | 20.53 | 1.00 | FAM-3981 | FAM14222 |  | MODERATE | ABC transporter ATP-binding protein | FAM14222p2\_000448 |
| 112 | 548547 | SNV | 20.66 | 1.00 | FAM-3981 | FAM14222 |  | MODERATE | chorismate-binding protein | FAM14222p2\_000450 |
| 113 | 700169 | INDEL | 26.37 | 1.10 | FAM-3981 | FAM14222 |  | n.a. | no hit | no hit |
| 114 | 717003 | SNV | 27.00 | 1.00 | FAM-3981 | FAM14222 |  | MODERATE | iron chelate uptake ABC transporter family permease subunit | FAM14222p2\_000617 |
| 115 | 771619 | SNV | 29.06 | 1.00 | FAM-3981 | FAM14222 |  | LOW | Type 1 glutamine amidotransferase-like domain-containing protein | FAM14222p2\_000667 |
| 116 | 780356 | SNV | 29.39 | 1.00 | FAM-3981 | FAM14222 |  | LOW | hypothetical protein | FAM14222p2\_000674 |
| 117 | 881076 | INDEL | 33.18 | 1.10 | FAM-3981 | FAM14222 |  | HIGH | dihydroxyacetone kinase subunit DhaL | FAM14222p2\_000767 |
| 118 | 881077 | INDEL | 33.18 | 1.10 | FAM-3981 | FAM14222 |  | MODERATE | dihydroxyacetone kinase subunit DhaL | FAM14222p2\_000767 |
| 119 | 881079 | INDEL | 33.18 | 1.10 | FAM-3981 | FAM14222 |  | MODERATE | dihydroxyacetone kinase subunit DhaL | FAM14222p2\_000767 |
| 120 | 906325 | SNV | 34.13 | 1.00 | FAM-3981 | FAM14222 |  | MODERATE | hypothetical protein | FAM14222p2\_000784 |
| 121 | 977165 | SNV | 36.80 | 1.00 | FAM-3981 | FAM14222 |  | LOW | PhoH family protein | FAM14222p2\_000848 |
| 122 | 1030213 | SNV | 38.80 | 1.00 | FAM-3981 | FAM14222 |  | LOW | VWA domain-containing protein | FAM14222p2\_000903 |
| 123 | 1046517 | INDEL | 39.41 | 1.10 | FAM-3981 | FAM14222 |  | n.a. | no hit | no hit |
| 124 | 1151190 | SNV | 43.35 | 1.00 | FAM-3981 | FAM14222 |  | n.a. | no hit | no hit |
| 125 | 1249699 | SNV | 47.06 | 1.00 | FAM-3981 | FAM14222 |  | MODERATE | methionyl-tRNA formyltransferase | FAM14222p2\_001100 |
| 126 | 1325286 | SNV | 49.91 | 1.00 | FAM-3981 | FAM14222 |  | MODERATE | L-threonylcarbamoyladenylate synthase | FAM14222p2\_001162 |
| 127 | 1376628 | SNV | 51.84 | 1.00 | FAM-3981 | FAM14222 |  | MODERATE | aconitate hydratase AcnA | FAM14222p2\_001216 |
| 128 | 1386059 | SNV | 52.20 | 1.00 | FAM-3981 | FAM14222 |  | MODERATE | TRAM domain-containing protein | FAM14222p2\_001224 |
| 129 | 1465456 | INDEL | 55.19 | 1.10 | FAM-3981 | FAM14222 |  | HIGH | citramalate synthase | FAM14222p2\_001286 |
| 130 | 1528357 | SNV | 57.56 | 1.00 | FAM-3981 | FAM14222 |  | MODERATE | ATP-dependent zinc metalloprotease FtsH | FAM14222p2\_001346 |
| 131 | 1555515 | SNV | 58.58 | 1.00 | FAM-3981 | FAM14222 |  | MODERATE | chromosome segregation protein SMC | FAM14222p2\_001376 |
| 132 | 1555894 | SNV | 58.59 | 1.00 | FAM-3981 | FAM14222 |  | MODERATE | chromosome segregation protein SMC | FAM14222p2\_001376 |
| 133 | 1561287 | INDEL | 58.80 | 1.10 | FAM-3981 | FAM14222 |  | n.a. | no hit | no hit |
| 134 | 1563365 | INDEL | 58.88 | 1.10 | FAM-3981 | FAM14222 |  | HIGH | pyridoxamine 5'-phosphate oxidase family protein | FAM14222p2\_001384 |
| 135 | 1563448 | INDEL | 58.88 | 1.10 | FAM-3981 | FAM14222 |  | HIGH | pyridoxamine 5'-phosphate oxidase family protein | FAM14222p2\_001384 |
| 136 | 1563458 | INDEL | 58.88 | 1.10 | FAM-3981 | FAM14222 |  | HIGH | pyridoxamine 5'-phosphate oxidase family protein | FAM14222p2\_001384 |
| 137 | 1563690 | INDEL | 58.89 | 1.10 | FAM-3981 | FAM14222 |  | HIGH | hypothetical protein | FAM14222p2\_001385 |
| 138 | 1563764 | INDEL | 58.89 | 1.10 | FAM-3981 | FAM14222 |  | MODERATE | hypothetical protein | FAM14222p2\_001385 |
| 139 | 1563766 | INDEL | 58.89 | 1.10 | FAM-3981 | FAM14222 |  | HIGH | hypothetical protein | FAM14222p2\_001385 |
| 140 | 1563767 | INDEL | 58.89 | 1.10 | FAM-3981 | FAM14222 |  | HIGH | hypothetical protein | FAM14222p2\_001385 |
| 141 | 1563769 | INDEL | 58.89 | 1.10 | FAM-3981 | FAM14222 |  | HIGH | hypothetical protein | FAM14222p2\_001385 |
| 142 | 1563770 | INDEL | 58.89 | 1.10 | FAM-3981 | FAM14222 |  | HIGH | hypothetical protein | FAM14222p2\_001385 |
| 143 | 1563853 | INDEL | 58.89 | 1.10 | FAM-3981 | FAM14222 |  | HIGH | ATP-dependent DNA helicase RecG | FAM14222p2\_001386 |
| 144 | 1583641 | SNV | 59.64 | 1.00 | FAM-3981 | FAM14222 |  | n.a. | no hit | no hit |
| 145 | 1676006 | SNV | 63.12 | 1.00 | FAM-3981 | FAM14222 |  | MODERATE | UDP-N-acetylmuramoyl-L-alanyl-D-glutamate--2%2C6-diaminopimelate ligase | FAM14222p2\_001489 |
| 146 | 1697144 | INDEL | 63.91 | 1.10 | FAM-3981 | FAM14222 |  | n.a. | 1-acyl-sn-glycerol-3-phosphate acyltransferase | FAM14222p2\_001506 |
| 147 | 1729970 | SNV | 65.15 | 1.00 | FAM-3981 | FAM14222 |  | LOW | DUF2510 domain-containing protein | FAM14222p2\_001537 |
| 148 | 1786392 | SNV | 67.27 | 1.00 | FAM-3981 | FAM14222 |  | MODERATE | thiazole synthase | FAM14222p2\_001591 |
| 149 | 1791074 | INDEL | 67.45 | 1.10 | FAM-3981 | FAM14222 |  | n.a. | no hit | no hit |
| 150 | 1829437 | SNV | 68.90 | 1.00 | FAM-3981 | FAM14222 |  | LOW | AI-2E family transporter | FAM14222p2\_001633 |
| 151 | 1859420 | SNV | 70.02 | 1.00 | FAM-3981 | FAM14222 |  | MODERATE | transcription-repair coupling factor | FAM14222p2\_001664 |
| 152 | 1916193 | SNV | 72.16 | 1.00 | FAM-3981 | FAM14222 |  | MODERATE | conjugal transfer protein TrbL | FAM14222p2\_001719 |
| 153 | 1944789 | SNV | 73.24 | 1.00 | FAM-3981 | FAM14222 |  | LOW | NCS2 family permease | FAM14222p2\_001755 |
| 154 | 1953368 | SNV | 73.56 | 1.00 | FAM-3981 | FAM14222 |  | n.a. | no hit | no hit |
| 155 | 1974195 | INDEL | 74.35 | 1.10 | FAM-3981 | FAM14222 |  | HIGH | NAD-binding protein | FAM14222p2\_001776 |
| 156 | 1976156 | SNV | 74.42 | 1.00 | FAM-3981 | FAM14222 |  | MODERATE | DUF6394 family protein | FAM14222p2\_001777 |
| 157 | 1977768 | SNV | 74.48 | 1.00 | FAM-3981 | FAM14222 |  | LOW | VTT domain-containing protein | FAM14222p2\_001779 |
| 158 | 1984893 | SNV | 74.75 | 1.00 | FAM-3981 | FAM14222 |  | MODERATE | DUF4032 domain-containing protein | FAM14222p2\_001784 |
| 159 | 1986985 | SNV | 74.83 | 1.00 | FAM-3981 | FAM14222 |  | MODERATE | lactate utilization protein B | FAM14222p2\_001787 |
| 160 | 2006343 | SNV | 75.56 | 1.00 | FAM-3981 | FAM14222 |  | LOW | phosphoglyceromutase | FAM14222p2\_001803 |
| 161 | 2012668 | SNV | 75.80 | 1.00 | FAM-3981 | FAM14222 |  | n.a. | no hit | no hit |
| 162 | 2018373 | SNV | 76.01 | 1.00 | FAM-3981 | FAM14222 |  | LOW | ferrochelatase | FAM14222p2\_001814 |
| 163 | 2046638 | SNV | 77.08 | 1.00 | FAM-3981 | FAM14222 |  | MODERATE | zinc-binding dehydrogenase | FAM14222p2\_001836 |
| 164 | 2065575 | SNV | 77.79 | 1.00 | FAM-3981 | FAM14222 |  | n.a. | no hit | no hit |
| 165 | 2086097 | SNV | 78.56 | 1.00 | FAM-3981 | FAM14222 |  | MODERATE | copper-translocating P-type ATPase | FAM14222p2\_001871 |
| 166 | 2122055 | SNV | 79.92 | 1.00 | FAM-3981 | FAM14222 |  | n.a. | no hit | no hit |
| 167 | 2180719 | SNV | 82.12 | 1.00 | FAM-3981 | FAM14222 |  | LOW | D-alanyl-D-alanine carboxypeptidase/D-alanyl-D-alanine-endopeptidase | FAM14222p2\_001957 |
| 168 | 2183926 | SNV | 82.25 | 1.00 | FAM-3981 | FAM14222 |  | LOW | histidine phosphatase family protein | FAM14222p2\_001960 |
| 169 | 2196694 | SNV | 82.73 | 1.00 | FAM-3981 | FAM14222 |  | LOW | hypothetical protein | FAM14222p2\_001972 |
| 170 | 2206179 | SNV | 83.08 | 1.00 | FAM-3981 | FAM14222 |  | MODERATE | methyltransferase | FAM14222p2\_001977 |
| 171 | 2258759 | SNV | 85.06 | 1.00 | FAM-3981 | FAM14222 |  | MODERATE | hypothetical protein | FAM14222p2\_002027 |
| 172 | 2289984 | SNV | 86.24 | 1.00 | FAM-3981 | FAM14222 |  | LOW | bifunctional diaminohydroxyphosphoribosylaminopyrimidine deaminase/5-amino-6-(5-phosphoribosylamino)uracil reductase RibD | FAM14222p2\_002050 |
| 173 | 2309729 | SNV | 86.98 | 1.00 | FAM-3981 | FAM14222 |  | MODERATE | nitrite/sulfite reductase | FAM14222p2\_002067 |
| 174 | 2325602 | SNV | 87.58 | 1.00 | FAM-3981 | FAM14222 |  | LOW | molybdate ABC transporter substrate-binding protein | FAM14222p2\_002077 |
| 175 | 2339188 | SNV | 88.09 | 1.00 | FAM-3981 | FAM14222 |  | LOW | molybdenum cofactor biosynthesis protein MoaE | FAM14222p2\_002087 |
| 176 | 2394795 | SNV | 90.19 | 1.00 | FAM-3981 | FAM14222 |  | n.a. | no hit | no hit |
| 177 | 2408238 | INDEL | 90.69 | 1.10 | FAM-3981 | FAM14222 |  | n.a. | no hit | no hit |
| 178 | 2603592 | SNV | 98.05 | 1.00 | FAM-3981 | FAM14222 |  | LOW | glucose PTS transporter subunit IIA | FAM14222p2\_002314 |
